# Observation for clinical effect of acupuncture combined with conventional therapy in the treatment of acne vulgaris

**DOI:** 10.1097/MD.0000000000019764

**Published:** 2020-05-01

**Authors:** Le Kou, Nan Yu, Junjie Ren, Bingyan Yang, Yun Tao

**Affiliations:** a Department of Dermatology, The Third People's Hospital of Ningxia; b Department of Dermatology, General Hospital of Ningxia Medical University; c Department of Tumor surgery, The First People's Hospital of Yinchuan; d Department of Dermatology, Huabei Petroleum General Hospital, China.

**Keywords:** acne vulgaris, acupuncture, protocol, randomized controlled trial

## Abstract

**Introduction:**

Acne vulgaris is a chronic inflammatory disease of the sebaceous glands that occurs in adolescent men and women. In recent years, the incidence of acne has increased year by year, so it is of great significance to find a precise and effective treatment and further explore its possible mechanism of action. The purpose of this study will be to explore a treatment method that has both traditional Chinese medicine characteristics and significant effects, and provides a higher level of evidence for acupuncture for acne vulgaris. It also provides patients with more treatment options.

**Methods/design:**

The study will be a randomized controlled trial divided into 2 parallel groups. This pragmatic randomized controlled trial will recruit 66 patients who are diagnosed with acne vulgaris. 30-minutes acupuncture sessions will be provided to patients assigned to the intervention group. All participants will continue to receive conventional treatment. The selection of outcomes will be evaluated by the skin lesions score scale.

**Discussion:**

This trial may provide evidence regarding the clinical effectiveness, safety, and cost-effectiveness of acupuncture for patients with acne vulgaris.

**Trial registration number:**

CTR2000030427

## 1 Introduction

Acne vulgaris is a chronic inflammatory disease of the sebaceous glands that occurs in adolescent men and women.^[1]^ Its occurrence is related to the gonad endocrine function in the human body. Once this function loses normal regulation, it will form chronic recurrent inflammation in the hair follicles and sebaceous glands of the human face.^[2]^ Acne vulgaris appears as acne, pimples, nodules, or cysts. According to relevant research data, the incidence of acne is about 20%, and nearly half of them occur in adolescents. In some regions, the incidence of adolescents exceeds 90%.^[3,4]^ Generally speaking, the incidence of men will not be lower than that of women. However, because women pay more attention to skin care, female patients often seek medical treatment in clinical investigations. With the improvement of living standards and economic conditions, people's requirements for beauty are getting higher and higher, and personal appearance is more and more important.^[5]^ Therefore, the prevention and treatment of acne is an urgent need of adolescents. Acne is a chronic inflammation in the type of pathological changes, and its lesions are in the hair follicles and secreting strong sebaceous glands.^[6]^ However, the most prone areas are the face and chest and back of the human body, because these areas are the areas where sebum secretion is more active. Acne develops further and can produce skin lesions like blackheads, pimples, cysts, and nodules.^[7,8]^ There is no name for acne in the classics of Chinese medicine, but there are many other names that are consistent with the pathology of acne. Generally speaking, these disease names are named according to the location of the disease and the characteristics of the skin lesions. For example, there are different disease names such as facial sores and acne. These disease names on the one hand reflect that the affected area of acne is generally on the face, and also reflect that the susceptible population of acne vulgaris is adolescents. According to the pathogenesis characteristics of the disease and the law of the development of the disease, traditional Chinese medicine (TCM) holds that the main pathogenesis of acne vulgaris is the accumulation of lung and stomach fever, or excessive thinking, discord between qi and blood, or imbalance of responsibility.

As for the treatment of acne vulgaris, mild acne is usually treated with topical drug smears, and moderate or severe acne is mostly treated with topical drug smears combined with antibiotics and retinoic acid drugs.^[9,10]^ Due to the long period of treatment for moderate to severe acne, patients need to take antibiotics for a long time, which greatly increases the incidence of adverse reactions such as nausea, vomiting, and dizziness, and it is easy to relapse after stopping the drug. TCM treats acne and adopts the principle of syndrome differentiation and treatment. Starting from the cause, the advantages are significant. Acupuncture, as a unique method of treating diseases in TCM, has the function of “internal disease and external treatment,” and can treat systemic diseases through the conduction of meridians and acupoints. In this study, the efficacy of acupuncture in the treatment of acne vulgaris will be observed through the establishment of commonly used western medicines as controls and clinical randomized controlled trials. We aim to explore the clinical efficacy of acupuncture in the treatment of acne and provide guidance for future clinical work and research work.

## 2 Methods/design

### 2.1 Study design and settings

**Figure 1:**
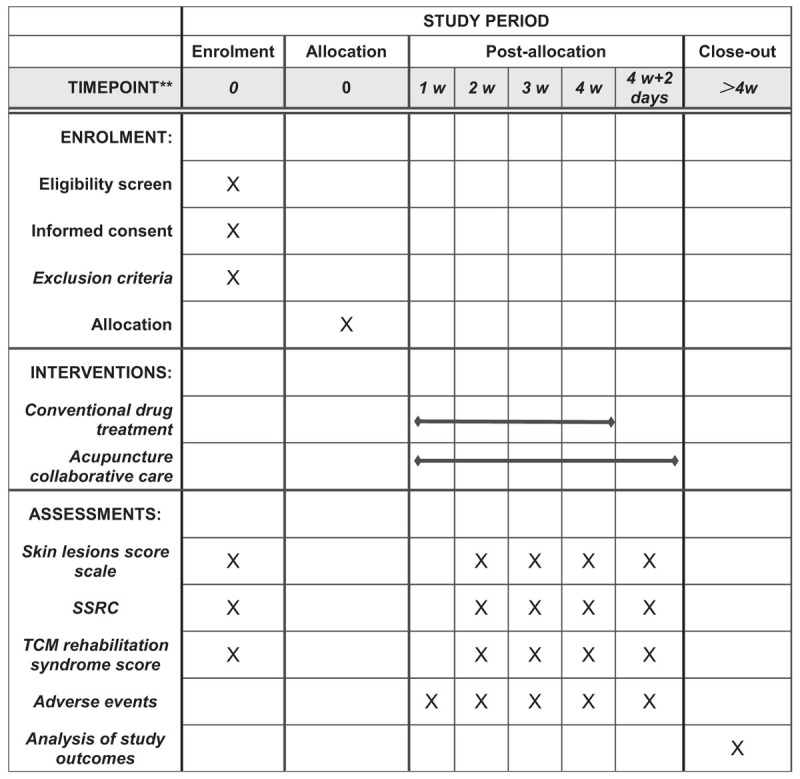
SPIRIT figure for the schedule of enrollment, interventions, and assessments. Abbreviations: SSRC = systemic symptom recording scale, SPIRIT = Standard Protocol Items: Recommendations for Interventional Trials, TCM = traditional Chinese medicine.

The study will be a randomized controlled trial divided into 2 parallel groups. This protocol is based on the Standard Protocol Project: Recommended Guidelines for Interventional Trials. If they agree, they will sign an informed consent form. Following the schedule described in Figure 1, only participants who read and agreed to the agreement and signed informed consent will be allowed to participate in the study. After patients were included in this clinical observation, the research guidelines of randomized controlled trials will be strictly followed, and the randomized number table method are going to be used to divide the admitted patients into test groups and control groups on average.

### 2.2 Participants

**Figure 2:**
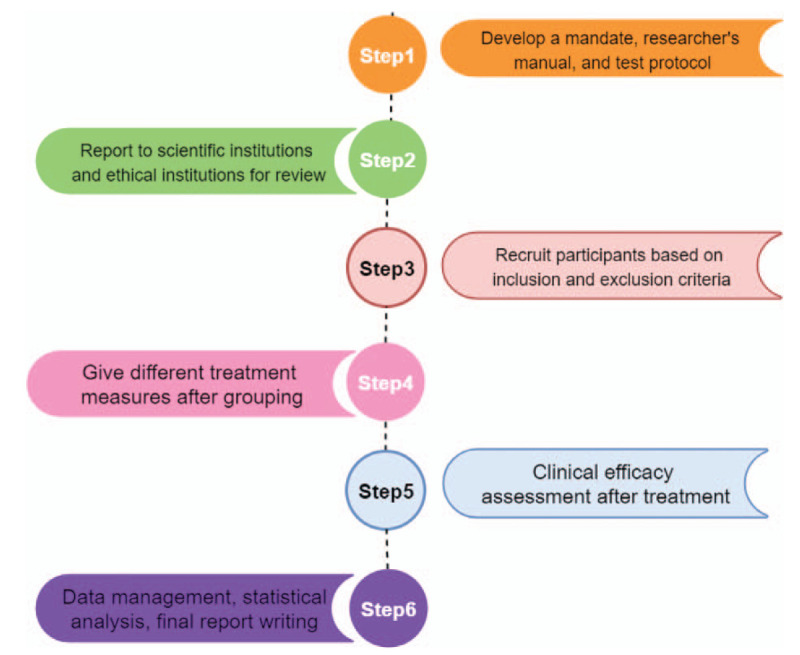
Study design flow chart.

All patients with acne vulgaris included in this clinical study will be recruited at the Third People's Hospital of Ningxia (Ningxia, China) acupuncture clinic and dermatology clinic. Case recruitment is initially scheduled for July 2020 to July 2021, with a total of 60 cases included (Fig. 2).

#### 2.2.1 Diagnostic criteria

The diagnostic criteria for western medicine used in this clinical research project are strictly in accordance with the relevant descriptions in the textbook “*Clinical Dermatology*” (editor: Zhao Zhan). The main points of diagnosis are as follows:

- ① The age of onset is young people, male, or female. The most prone areas are the cheeks and forehead, followed by some areas with strong sebum secretion, such as the front chest, back, and shoulders.
- ② The lesions show skin-like changes, usually pimples, blackheads, acne, knots, nodules, cysts, and scars. These skin lesions are generally symmetrical in the affected area, and are usually accompanied by strong sebum secretion, a slow and persistent condition.

Acne Severity Grading Standards: The acne grading standards used in this clinical research project are derived from the grading standards established by Pillsbury. The classification criteria are as follows:

- ① Grade I (mild): most of the types of skin lesions are blackheads, which do not appear intensively or intermittently, and are accompanied by a small amount of inflammatory pimples.
- ② Grade II (moderate): most of the types of skin lesions are acne. The lesions are mainly concentrated on the face, accompanied by a moderate number of pimples and acne that may develop further.
- ③ Grade III (Severe): the type of skin lesions is mainly inflammatory pimples and acne. The main lesions are on the face, neck, chest, and back, with a small number of nodules (less than 3). The number is around 51 to 100.
- ④ Grade IV (severe): the type of skin lesions is mainly inflammatory pimples that may develop further, accompanied by more nodules and cysts (greater than 3), the number of skin lesions is greater than 100, and the probability is greater scar.

#### 2.2.2 Inclusion criteria

- ① Patients who meet the above-mentioned diagnostic criteria for acne vulgaris; aged between 16 and 40 years; the severity of acne reaches grade II or above.
- ② Did not take any drugs related to the treatment of acne within 1 month before receiving this clinical research protocol.
- ③ Do not take antibiotics, glucocorticoids or retinoic acid drugs within 2 weeks.
- ④ I have not received any other topical medications related to acne within a week.
- ⑤ Those who have no objection to the informed consent of this clinical research trial and agree to be observed and signed the informed consent form.

#### 2.2.3 Exclusion criteria

Patients will be excluded if they meet the following criteria:

- ① The incidence of acne is classified as patients with nodular acne, agglomerated acne, scarring acne, fulminant acne, and premenstrual acne.
- ② During the observation period of this clinical study, patients who are unavoidably exposed to direct sunlight or receive high-intensity ultraviolet rays are unavoidable.
- ③ Acne caused by long-term exposure to specific chemicals or acne caused by long-term use of glucocorticoids.
- ④ Those who are allergic or scarred, and patients who disagree with the treatment plan used in this clinical research.
- ⑤ Patients with severe cardiovascular and cerebrovascular diseases, cardiopulmonary insufficiency, liver and kidney dysfunction, and severe infections;
- ⑥ Women during pregnancy or lactation.

#### 2.2.4 Elimination, shedding, and abort test criteria

Researchers participating in clinical trials should carefully record the reasons for the suspension of the trial and the relationship with the clinical trial. It is necessary to clearly record the unwillingness of the subjects to continue the clinical trials, put forward the reasons for withdrawing from the clinical trials, and record the evaluation indicators at the time of discontinuation in detail.

- ① Patients with incomplete data due to various reasons during the clinical observation process, which could not accurately determine the curative effect.
- ② Patients who withdrew from the clinical observation treatment plan or did not cooperate with staff during the treatment for various reasons.
- ③ Patients with severe adverse reactions or treatment accidents during the treatment process of this clinical observation.
- ④ Patients who used other treatments related to acne without consent during the treatment process of this clinical observation.

### 2.3 Interventions

The intervention group and the control group were treated according to the following schemes, respectively. At the same time, the 2 groups of patients should be cleaned daily, gentle facial cleanser to wash excess facial oil, and avoid direct sunlight.

Control group: The control group will be given topical AA cream. After instructing the patient to clean the face by themselves, use a clean cotton swab to apply an appropriate amount of vitamin A acid cream to the redness and swelling of the skin, and apply it once a day before going to bed at night. The total course of treatment is 4 weeks.

Intervention group: Patients in the intervention group will be given the same treatment measures as those in the control group, while undergoing acupuncture treatment. Differentiated acupoints and local points of skin lesions will be used. The syndrome of wind-heat will be taken from *Hegu, Quchi, Chize, Weizhong, Feishu, Fengmen*, and Damp-heat syndrome will be taken from *Hegu, Quchi, Zusanli, Sanyinjiao and Yinlingquan*. The patients are going to be placed in the lateral position, and the acupoints were routinely disinfected with 75% ethanol. The chest, abdomen and limbs were acupunctured with a 1.5-inch needle, and the face was punctured with a 1-inch needle. The needle-needle method is used to lift the needle, and the needle is twisted and twisted. Based on the patient's local feeling of soreness and numbness, the needle will be left for 30 minutes, and the needles are going to be given every 10 minutes during this period. Acupuncture treatment is once every other day, 10 treatments are 1 course, and 3 courses are treated.

### 2.4 Outcome measures

#### 2.4.1 Primary outcome measures

We will use the skin lesions score scale as the primary outcome indicator. This scoring table was developed in accordance with the guidelines of the latest version of the “Guidelines for Clinical Research of New Chinese Medicines.” This score sheet is an evaluation index for the main symptoms of acne, and comprehensively evaluates the main efficacy of each treatment plan. The scoring items of the scoring table are to evaluate the amount and distribution of skin lesions such as pimples, nodules, and cysts. There are 4 main items to be scored, namely the type of skin lesions, the number of skin lesions, the degree of swelling and rupture, and the color of the skin lesions. Each item is divided into 4 severity levels, which are rated as 0 points, 2 points, 4 points, and 6 points in turn, and the total score is finally calculated.

#### 2.4.2 Secondary outcome measures

We will use the systemic symptom recording scale as the secondary outcome measure. This record scoring table is based on TCM-related symptoms of acne. It reflects the changes in the symptoms of acne patients after various treatments, and it is of auxiliary evaluation significance. Evaluation content includes complexion, emotion, urination, stool, sleep, appetite, menstruation (female patients only), etc. The score is divided into 2 levels, which is 0 for normal and 1 for abnormal. The above 2 scoring tables were evaluated every 2 weeks before and during the treatment, that is, a total of 3 evaluations. Observe the therapeutic effect of each treatment scheme on the condition of acne as a whole.

### 2.5 Sample size calculation

This study is a small sample clinical trial. According to statistical requirements, there are at least 30 cases in each group. Considering that the drop-out rate does not exceed 20%, a total of 66 cases were collected, of which 30 were in the intervention group and the other in the control group.

### 2.6 Randomization and blinding

All 60 patients with acne vulgaris that met the research criteria will be divided into intervention group and control group, each with 33 cases. All patients included in this clinical observation will be given numbers from 1 to 60 according to the time of inclusion. Then abide by the idea of random method, the computer software generates 60 random numbers, and the 60 random numbers correspond to the patient number one by one. The random numbers assigned to the patients after the division are going to be divided by 2. If the random number is divisible, the patient will be assigned to the intervention group. If the random number is not divisible, it will be assigned to the control group (that is, the random number is incorporated into the experimental group and the odd number is incorporated into the control group). Until 1 group was full, the remaining patients were assigned to the other group.

### 2.7 Statistical analysis.

SPSS for windows 24.0 statistical analysis software will be used for calculation, and normality test and homogeneity test of variance will be performed on each group of data. For measurement data in which the data conforms to the normal distribution, we will use the mean ± standard deviation. For non-normally distributed measurement data, the median ± quartile interval is used. General data comparison between the 2 groups using independent sample *t* test. Comparisons before and after treatment will be performed using *t* test for paired data. One-way analysis of variance will be used for comparison between groups. X^2^ test will be used for count data, and nonparametric rank sum test will be used for rank data. All statistical tests are two-sided. *P* < .05 indicates a significant difference.

### 2.8 Data management

Information obtained from the evaluation of each participant will be recorded on a paper printout. The information will then be handwritten on a paper document case report form and entered into an Excel file for future statistical analyses. In accordance with the Personal Information Protection Act, the names of all participants will not be disclosed, and a unique identifier number given during the trial will be used to identify participants. All of the participants will be informed that the clinical data obtained in the trial will be stored in a computer and will be handled with confidentiality. The participants’ written consent will be stored by the principal investigator.

### 2.9 Ethics

The study protocol is going to be approved by the ethics committee of the Third People's Hospital of Ningxia. The study will be conducted under the Declaration of Helsinki principles, as well as following the norms of good clinical practice. Recruitment of patients has not started in this study. The study plan will be submitted to the ethics committee of the Third People's Hospital of Ningxia for review. We will not start recruiting participants without the consent of the ethics committee. The protocol of this study has been registered in the Chinese Clinical Trial Registry with the number ChiCTR2000030427.

## 3 Discussion

In recent years, with the improvement of living standards and the improvement of dietary conditions, the incidence of patients has increased year by year, and the age of onset has gradually increased.^[11]^ Data from a census study in the United States show that the incidence of acne is as high as 85% in people aged 12 to 24. A survey of students aged 16 to 18 in an Australian region found that 97.8% of boys and 89.8% of girls have face and neck acne.^[12]^ 17% of them are moderate and severe. Acne not only damages the appearance of patients, but also causes bad emotions such as anxiety or depression due to impaired appearance, which seriously affects the physical and mental health of patients.^[13]^ Regarding the pathogenesis of acne, modern medicine believes that it is mainly related to endocrine factors, immune factors, abnormalities of the hair follicle sebaceous glands, and mass reproduction of microorganisms.

Recent studies have shown that acupuncture can play a key role in the pathophysiology of acne. It can directly stimulate acne lesions, act on hair follicles, kill microorganisms, and then promote the subsidence of inflammation.^[14,15]^ In addition, acupuncture can quickly eliminate or relieve local tissue edema, congestion, exudation, adhesions, and other pathological changes, so that the damaged tissue can be repaired again. The mechanism may be related to the following aspects:

1. The white blood cell value changes significantly before and after acupuncture treatment. The white blood cell count of patients treated with acupuncture was significantly higher, indicating that acupuncture can promote the proliferation of white blood cells, thereby promoting the absorption of inflammation and playing a role in anti-inflammatory and anti-infection.^[16]^ Acupuncture has the function of promoting the absorption of chronic inflammation, which can destroy the diseased tissue and stimulate the body's absorption of necrotic tissue.
2. Promote blood circulation: There are a variety of abnormalities in blood rheology in acne patients, which can cause local microcirculation disorders and lead to local skin lesions.^[17,18]^ Clinical studies in recent years have shown that some changes in human hemodynamics occur after acupuncture treatment, mainly manifested by an accelerated hemodynamic speed.^[19]^ We consider that the reason may be that the microcirculation has changed, which directly affects the blood flow velocity.
3. Acupuncture can directly destroy sebaceous gland cells, reduce excessive secretion of sebum, and change the living environment of microorganisms in Maoxiang. At the same time, it creates a pathway for the discharge of inflammatory substances, thereby promoting the absorption of inflammation and accelerating local blood circulation.^[20,21]^ It can improve the body's metabolic level and improve the state of the body. In recent years, the incidence of acne has increased year by year, so it is of great significance to find a precise and effective treatment and further explore its possible mechanism of action. The purpose of this study will be to explore a treatment method that has both TCM characteristics and significant effects, and provides a higher level of evidence for acupuncture for acne vulgaris. It also provides patients with more treatment options.

## Acknowledgments

The authors would like to thank all the trial participants. The authors are grateful for the support for this study: trial coordinating team, surgical staff, nurses, and research departments.

## Author contributions

KL, NY and JJR designed the study protocol and drafted the manuscript. BYY reviewed the study protocol and drafted the manuscript. YT is responsible for the statistical design and analysis as trial statistician. All authors carefully read and approved the final version of the manuscript. KL participated in the design and coordination of the study. All authors read and approved the final manuscript.
